# Long-Term Supplementation of GABA Regulates Growth, Food Intake, Locomotion, and Lipid Metabolism by Increasing Ghrelin and Growth Hormone in Adolescent Mice

**DOI:** 10.3390/nu17101634

**Published:** 2025-05-10

**Authors:** Rafael Begazo-Jimenez, Amelia Yu, Robert Gros, Wei-Yang Lu

**Affiliations:** 1 Neuroscience Graduate Program, Schulich School of Medicine and Dentistry, University of Western Ontario, London, ON N6A 3K7, Canada; rbegazoj@uwo.ca; 2 Department of Physiology and Pharmacology, Schulich School of Medicine and Dentistry, University of Western Ontario, London, ON N6A 3K7, Canada; ameliayu0825@gmail.com; 3 Department of Medicine, Schulich School of Medicine and Dentistry, University of Western Ontario, London, ON N6A 3K7, Canada

**Keywords:** GABA, adolescent development, lipid metabolism, growth hormone, ghrelin

## Abstract

**Background/Objectives**: The amino acid γ-aminobutyric acid (GABA) is the primary neurotransmitter in the central nervous system (CNS) and acts as an autocrine and/or paracrine signaling molecule in various types of non-neuronal cells. On the other hand, GABA is a nutrient found in a variety of foods and is marketed as a health supplement based on a growing number of studies reporting health benefits in humans and recuperations in animal models of diseases. The present study sought to examine whether supplementation of GABA to young mice regulates their growth as well as glucose and lipid metabolism during physiological adolescence. **Methods**: Mice were supplemented with GABA over a 16-week period with subsequent anthropometric, metabolic, and endocrine measurements. **Results**: Results showed that 16-week oral supplementation of GABA increased food consumption and body length while attenuating weight gain in male mice but not females. In addition, GABA treatment lowered the index of body fat (Lee index) and increased the expression of lipolytic enzymes in adipose and liver tissues of male mice without affecting blood glucose levels. Remarkably, supplementation of GABA significantly increased the protein expression of growth hormone (GH) in the pituitary gland of both male and female mice. However, it only substantially increased GH levels in the sera of male mice but not females. Moreover, GABA significantly increased the expression of the GH secretagogue peptide ghrelin in the stomachs of male mice only. **Conclusions**: Together these novel findings suggest that long-term GABA supplementation fundamentally influences the growth and lipid metabolism of males during adolescent development by stimulating ghrelin–GH production and secretion. The mechanisms of GABA-induced sex-dependent upregulations of ghrelin and GH, as well as lipid metabolism in adolescence, await further studies.

## 1. Introduction

Amino acids are one of the major nutrients that critically regulate key pathways necessary for metabolic maintenance, growth, reproduction, and immunity of the body [1]. The non-protein amino acid γ-aminobutyric acid (GABA) is the primary inhibitory neurotransmitter in the mature central nervous system (CNS) of mammalians, and it also acts as a “trophic factor” during development to influence neuronal proliferation, differentiation, and synapse maturation [2]. Importantly, numerous studies showed that in the body of mammalians, GABA is produced as an autocrine and/or paracrine signaling molecule by various types of non-neuronal cells including lymphocytes [3], macrophages [4], epithelial cells [5,6], hepatocytes [7], endocrine cells [8,9,10], and adipocytes [11,12], critically regulating systemic immunity and metabolism.

GABA, as a nutrient, exists in various foods such as wheat, vegetables, fruits, and fermented dairies [13,14]. GABA is also marketed as a health supplement based on a growing number of studies reporting health benefits in humans and improvements in animal models of diseases. In addition, there is growing interest in GABA supplementation among the livestock and poultry industries in which it has shown potential benefits on feeding, growth, immunity, and other markers of health in animals [15,16,17,18]. Importantly, various studies report that oral administration of GABA to high-fat-diet-treated rodents can suppress weight gain and inflammation, primarily by regulating the metabolism of glucose and lipids [19,20,21], as well as by regulating the phenotypes and metabolic activity of macrophages in the adipose tissues [22] and liver [23]. These studies significantly advance the current understanding of the effects of GABA on the regulation of metabolism under certain disease conditions.

Adolescence in mice refers to the developmental phase starting at weaning and ending at sexual maturity [24]. It is a nutrition-sensitive phase for growth, in which the benefits of adequate nutrition extend to many physiological systems. The nutritional effects on adolescent development extend beyond body growth, because growth has profound consequences on an individual’s development and health in later life [25]. Despite the increasing amount of research on GABA supplementation in the context of obesity and metabolic disease, the role of GABA in the regulation of growth and metabolism during juvenile/adolescent development is not well understood. The present study sought to examine whether oral supplementation of GABA regulates growth, as well as glucose and lipid metabolism, during adolescent development of mice under the condition of a calorie-controlled “standard” chow diet and, if so, identify the key cellular mechanisms through which GABA exerts its action.

## 2. Materials and Methods

### 2.1. Animals and Oral GABA Treatment

A total of 104 (52 male, 52 female) C57BL/6J weaned (23–30 days old) mice were housed in a temperature- and humidity-controlled environment with a 12 h light/dark cycle at a room temperature of 22 ± 2 °C and a humidity of 55 ± 5%. Male and female mice were accommodated separately and housed 4 per cage. Mice were randomly grouped for oral (ad libitum) GABA treatment (32 males and 32 females) and as controls (20 males and 20 females). While control mice had plain drinking water, mice in the treatment groups were provided with drinking water containing GABA (Sigma-Aldrich, St. Louis, MO, USA) at a concentration of 2 mg/mL. Drinking water was replaced weekly over a 16-week period of GABA treatment. Investigators were responsible for preparing the GABA water weekly and providing it to the treatment group, as well as for performing analyses on the collected samples; therefore, they were aware of the control and treatment group allocation at all stages of the experiment. Given that post-weaning nutrition determines the metabolic risk of mice exposed to overnutrition during early life stages [26], the mice in the present study were fed with a Teklad 2018 diet that contained low-fat (6%), as well as low alfalfa and soybean meal, which are significant sources of isoflavones/phytoestrogen, thus minimizing the potential effects on hormonal balance, inflammation [27], and lipid metabolism [28]. All experimental procedures were performed in accordance with the Animal Use Protocol #2022-125, which was approved by Animal Care and Veterinary Services at the University of Western Ontario.

### 2.2. Measurements of Daily Water and Food Consumption

Averaged daily water and food consumption per mouse were measured and calculated weekly for 16 weeks. Prior to GABA treatment, average food and water intake per day in all groups was measured for 7 days to obtain baseline water and food consumption values. Water and food intake was then continuously monitored during the GABA treatment period by weighing the food and measuring the water volume before and after each weekly recording period. During each measurement, smaller pellets of food were removed to prevent them from falling through the feeding cage. The bedding of the mouse housing was also manually sifted to account for any small pellets or crumbs that may have fallen through the feeding cage. These crumbs of food were then added to the measurement of the leftover amount. Food was replenished weekly and controlled so that all cages had the same starting amount of food each week. Average food intake per animal was calculated for the 16-week treatment period by averaging the weekly cage-level measurements during the treatment period and dividing that value by 4 mice per cage.

### 2.3. Anthropometric Measurements

At the time of anthropometric measurements, the general conditions of the mice were closely watched. Notably, the treated mice were physically active, and no gastrointestinal abnormalities were observed. Bodyweight (g) and nasoanal length (mm) were measured before and after the treatment period. The final bodyweight and nasoanal length values were used to calculate an indicator of rodent body fat content, the “Lee index” [29], by using the following formula:

Lee Index = [(weight in g) 0.33/nasoanal length (cm)] × 1000.

### 2.4. Metabolic Assessments

Oxygen consumption, carbon dioxide production, respiratory exchange ratio (RER), food and water intake, physical activity, and sleep were measured in male and female mice using the Comprehensive Lab Animal Monitoring System (CLAMS) interfaced with Oxymax software V4.75 (Columbus Instruments, Columbus, OH, USA), as previously described [30]. Briefly, mice were individually housed in metabolic chambers maintained at 24 ± 1 °C and given ad libitum access to powdered standard rodent chow and water. All the measurements were taken every 10 min for 24 h (12 h light/12 h dark) after a 16 h habituation period in the individual metabolic chambers. Total activity, ambulatory activity, and sleep (periods of inactivity) were obtained using the Opto-M3 Activity Monitor and Oxymax software algorithms (Columbus Instruments), as previously described in detail [30].

### 2.5. Measurement of Blood Glucose

As previously reported, GABA treatment in a mouse model of type 1 diabetes restored levels of insulin in pancreatic beta cells [10]. Therefore, after 16 weeks of GABA supplementation, the blood glucose concentrations of mice were measured via tail puncture using a glucose meter (OneTouch Ultra2, LifeScan, Wayne, PA, USA).

### 2.6. Serum Collection and GH Assay

Sixteen weeks after GABA treatment, all test mice were deeply anesthetized using isoflurane. Blood was collected by cardiac puncture under anesthesia and then serum was obtained and stored at −80 °C. Growth hormone (GH) concentration was measured later using the GH ELISA Kit (Invitrogen, Waltham, MA, USA, #KRC5311) following the manufacturer’s instructions.

### 2.7. Tissue Collection

After blood collection, the pancreas, liver, and gonadal fat were isolated and collected from all tested mice. In addition, the pituitary gland and hypothalamus were also collected, respectively, under a surgical microscope using previously reported procedures with modifications [31,32]. The collected tissues were either fixed in 4% paraformaldehyde or stored at −80 °C for later use.

### 2.8. Immunohistochemistry

After 48 h of fixation, tissues were placed in PBS and then were paraffin-embedded and sliced accordingly. As performed previously [10], pancreatic tissues were serially cut into 5 μm sections at 50 μm intervals. After serum blocking, tissue sections were incubated at 4 °C overnight with specific primary antibodies against insulin (Cell Signaling Technology, Danvers, MA, USA, #4590). After primary antibody fostering, sections were incubated with a Cy3- or FITC-conjugated secondary antibody. A section without a primary antibody was used as a negative control. Nuclei were stained with DAPI. Confocal microscopic images were taken from each section. Images were analyzed using Image-J V1.54k open-source software (National Institutes of Health, Bethesda, MD, USA), as previously described [6,33]. β-cell mass was calculated by multiplying the average insulin-positive area in relation to the whole pancreatic area with the pancreatic weight of the corresponding animal, as described [10,34]. To assess each of the immunostained proteins, we analyzed between 18 and 68 islets in pancreatic slices prepared from five or six control and GABA-treated mice. The areas of insulin-positive cells in the pancreatic islet were quantified as a measure of β-cell mass, respectively.

As previously described [31], coronal sections of the pituitaries were sliced at 4 μm thickness. Antigen retrieval was performed by immersion in sodium citrate buffer at 95 °C. Slides were permeabilized in 0.25% Triton-X solution for 5 min, blocked with 10% normal donkey serum, and subsequently double-stained with goat anti-GH (R&D Systems, Minneapolis, MN, USA, #AF1067) plus guinea pig anti-GABA_A_R alpha-1 subunit (Alamone, Jerusalem, Israel, #AGP-083) antibodies overnight. Slides were washed in PBS and then incubated with the corresponding secondary antibodies for 2 h. The nuclear marker, DAPI, was then incubated for 15 min prior to mounting the cover glass using Fluoromount-G (Electron Microscopy Solutions, Hatfield, PA, USA). Images of pituitary sections were acquired using a Nikon A1 series confocal laser microscope at 60× magnification using 1024-pixel resolution. Images were analyzed for immunofluorescence using ImageJ V1.54k. Cell area and perimeter were calculated using a combination of watershed sectioning and thresholding for automated cell segmentation and counting.

### 2.9. Western Blot

Dissected tissues were frozen at −80 °C. Samples were homogenized in radioimmunoprecipitation assay (RIPA) lysis buffer with 0.1% apoprotein and leupeptin. Tissue lysates were then centrifuged at 13,000 rpm for 30 min at 4 °C. Adipose tissue was processed differently, as previously described, with some modifications [35]. Briefly, adipose tissue was homogenized in RIPA buffer and immediately centrifuged at low speed (6000× *g*) for 15 min at 4 °C. The top lipid layer was then carefully removed from the samples using a pipette. The pellets were then re-suspended in the RIPA buffer with 1% triton and samples were incubated at 4 °C for 60 min. After incubation, samples were centrifuged at high speed (1200× *g*) for 15 min at 4 °C and the upper layer of lipid was again removed from the samples. These last steps were repeated 1–2 times until there was no visible lipid layer in the samples.

After collecting the supernatant, total protein content was measured by Bradford assay (Bio-Rad, Hercules, CA, USA, #5000006). Samples were prepared using 5× sample buffer, loaded to 15% SDS-PAGE gel for 2 h at 100 V, and then transferred to a polyvinylidene difluoride membrane for 2 h at 80 V. Membranes were blocked with 5% bovine serum albumin for 1.5 h before incubation with the goat anti-GH (R&D Systems, #AF1067), rabbit anti-Ghrelin (Abcam, Cambridge, UK, #ab129383), rabbit anti-GHS-R (Abcam, #ab85104), goat anti-IGF-1 (ThermoFisher, Waltham, MA, USA, #500-P157G), rabbit anti-HSL (Cell Signaling Technology, #4107), rabbit anti-*p*-HSL (Cell Signaling Technology, #4139), mouse anti-GAPDH (Abcam, #ab9482), or rabbit anti-pAKT(Thr308) (Cell Signaling Technology, #9275) antibody overnight at 4 °C, using the manufacturer’s recommendations for antibody dilution. The membranes were washed 3 times in Tris Buffered Saline with Tween 20 (TBS-T) and then incubated for 1.5 h in the appropriate horseradish peroxidase-conjugated secondary antibody at room temperature. The membrane was then incubated for 1 min in chemiluminescence substrate (ThermoFisher, #32106) and imaged using the Versa Dock 5000 MP system by Bio-Rad with Quantity One imaging software V4.6.9. Protein quantification analyses were performed using ImageJ V1.54k open-source software. All values were normalized to the GAPDH loading control prior to statistical analysis.

### 2.10. Statistical Analysis

Data were visually assessed for normality using Q-Q plots which showed approximate linearity with no major deviations from the expected normal distribution. Results were statistically analyzed via unpaired Student’s *t*-tests using GraphPad Prism 10. A *p*-value less than 0.05 was determined to indicate a significant difference between groups. All statistic values and figures were presented as means ± standard error of the mean (SEM).

## 3. Results

### 3.1. Effects of GABA on Food and Water Consumption

Changes in nutrient amino acid concentrations can influence cell metabolism, subsequently modulating feeding behavior, as well as water and food consumption [36]. Given that GABA regulates cellular metabolism via its receptors expressed by various types of cells and/or through the “GABA-shunt” [37], we examined whether ad libitum supplementation of GABA affected the daily food and water consumption of growing mice.

**Figure 1:**
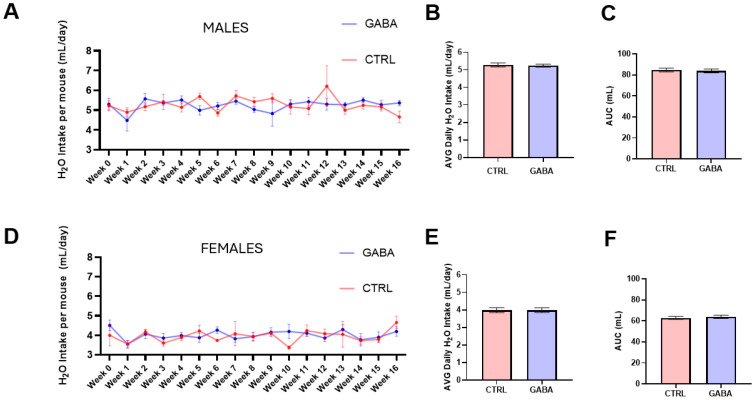
GABA supplementation did not change water consumption. Measurements were performed weekly per cage (12 controls and 20 GABA-treated mice per sex). (**A**) The volumes of weekly average water consumption. (**B**) The average daily water intake by GABA-treated male mice compared to controls (control: 5.28 ± 0.11 mL, n = 3 cages; GABA-treated: 5.25 ± 0.08 mL, n = 5). (**C**) Analysis of the area under the curve (AUC) of 16-week water consumption between the control and treated males (control: 84.75 ± 1.75 mL, n = 3; GABA-treated: 83.89 ± 1.8 mL, n = 5). (**D**) Volumes of weekly average water consumption by control and GABA-treated female mice. (**E**) Average daily water consumption between control and GABA-treated female mice (control: 3.98 ± 0.14 mL, n = 3; GABA-treated: 3.99 ± 0.14 mL, n = 5). (**F**) Analyses of AUC of 16-week water consumption between the control and treated females (control: 62.89 ± 1.47 mL, n = 3; GABA-treated: 63.95 ± 1.49 mL, n = 5).

During the 16-week treatment, both control and GABA-treated male and female mice were physically active and did not display gastrointestinal abnormalities. Our records showed that the volume of weekly average water consumption by male mice (Figure 1A) was slightly higher than that of female mice (Figure 1D). Specifically, the average daily water intake by GABA-treated male mice was comparable to that by the control males (Figure 1B). Likewise, the average daily water intake by the GABA-treated female mice was not significantly different from that of female controls (Figure 1E). Analysis of the area under the 16-week water-consumption curve revealed no significant differences in the total cumulative water intake between control and GABA-treated mice of both sexes (Figure 1C,F).

**Figure 2:**
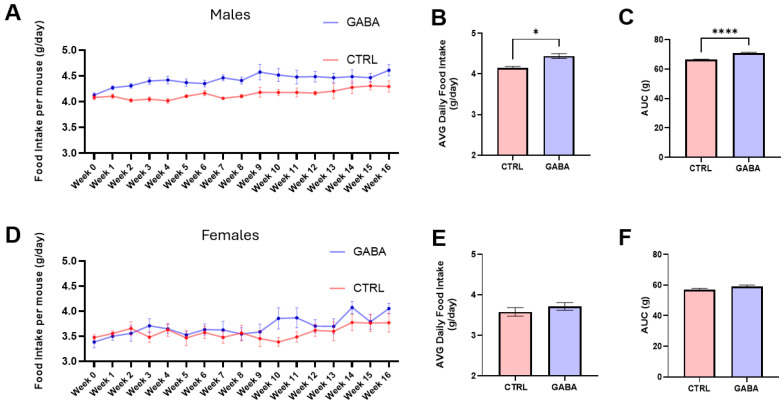
GABA supplementation increases food consumption in male mice. Measurements were performed weekly per cage in 12 controls and 20 GABA-treated mice per sex. (**A**) Longitudinal line plot of the average weekly food intake by control and GABA-treated males. (**B**) Average daily food intake of GABA-treated male mice compared to controls (control: 4.15 ± 0.04 g, n = 3 cages; GABA-treated: 4.44 ± 0.06 g, n = 5; *p* < 0.05). (**C**) Analyses of the AUC of the 16-week food intake between GABA-treated males and controls (control: 66.29 ± 0.35 g, n = 3; GABA-treated: 70.8 ± 0.59 g, n = 5; *p* < 0.0001). (**D**) Weekly food consumption by control and GABA-treated females during the 16-week treatment period. (**E**) The average daily food intake by GABA-treated females compared to controls (control: 3.58 ± 0.11 g, n = 3; GABA-treated: 3.71 ± 0.1 g, n = 5; *p* = 0.41). (**F**) AUC analysis of 16-week food intake between GABA-treated and control females (control: 57.13 ± 0.66 g, n = 3; GABA-treated: 59.05 ± 0.89 g, n = 5; *p* = 0.09). Statistical significance indicated as * *p* < 0.05, **** *p* < 0.0001.

As shown in Figure 2A, the weekly food intake by control males remained relatively stable, whereas the weekly food consumption by GABA-treated males increased gradually and significantly in the first 1–9 weeks, and these GABA-treated males kept consuming significantly more food compared to control males in the remaining treatment weeks. Additional analyses showed that over the 16-week period, the average daily food intake by GABA-treated male mice was significantly more (about 6.5% more) than that of control male mice (Figure 2B). Furthermore, analysis of the area under the 16-week food-consumption curve revealed a greater cumulative food intake over the treatment period in the GABA-treated males compared to controls (Figure 2C). In contrast, the weekly food consumptions by both control and GABA-treated females remained relatively stable and did not show significant differences during the 16-week treatment period (Figure 2D). Extended analyses revealed that over the whole treatment period, the average daily food intake (Figure 2E) and the cumulative food intake (Figure 2F) showed no significant differences between GABA-treated and control female mice.

### 3.2. Effects of GABA on Bodyweight, Nasoanal Length, and Lee Index

**Figure 3:**
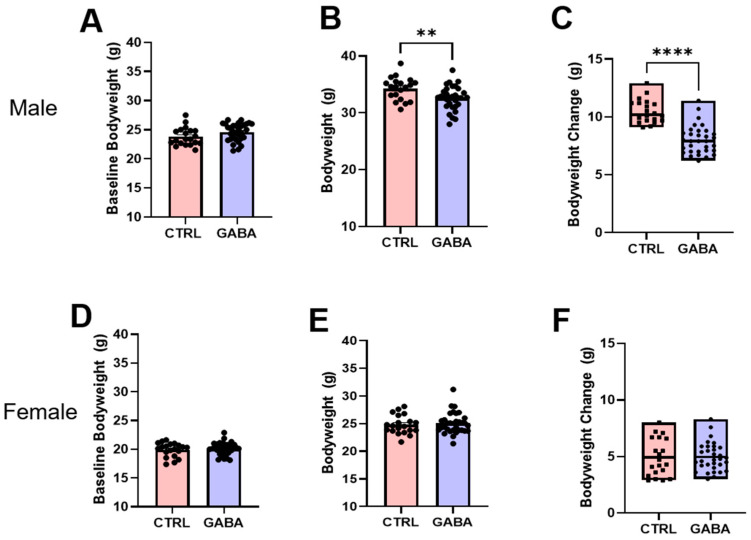
GABA supplementation reduces the bodyweight gain of male mice but not female mice. (**A**) Pre-treatment bodyweight of controls compared to GABA-treated males (control: 23.81 ± 0.34 g, n = 20; GABA-treated: 24.59 ± 0.26 g, n = 32; *p* = 0.07). (**B**) Post-treatment bodyweight of control and GABA-treated males (control: 34.29 ± 0.44 g, n = 20; GABA-treated: 32.62 ± 0.37 g, n = 32; *p* < 0.01). (**C**) Box plots of total bodyweight gain between control and GABA-treated males (control: 10.49 ± 0.23 g, n = 20; GABA-treated: 8.03 ± 0.22 g, n = 32; *p* < 0.0001). (**D**) Baseline bodyweight of the female controls compared to the GABA-treated mice (control: 19.9 ± 0.27 g, n = 20; GABA-treated: 20.11 ± 0.19 g, n = 32). (**E**) Post-treatment bodyweight of control and GABA-treated females (control: 24.82 ± 0.38 g, n = 20; GABA-treated: 25.08 ± 0.34 g, n = 32). (**F**) Bodyweight gain of control females versus the GABA-treated group (control: 4.92 ± 0.37 g, n = 20; GABA-treated: 4.97 ± 0.22 g, n = 32). Statistical significance indicated as ** *p* < 0.01, **** *p* < 0.0001.

We also examined the effects of GABA supplementation on bodyweight and nasoanal length, thereby studying the influence of GABA on the Lee index of male and female mice. As shown in Figure 3A, the pre-experimental average bodyweight of control male mice was similar to that of the GABA-treated males. At the end of the 16-week treatment, the bodyweight of control males was significantly heavier than that of GABA-treated males (Figure 3B). Extended analyses showed that over the entire treatment period, the bodyweight gain of GABA-treated males was about 23% less than that in control males (Figure 3C). The baseline bodyweight of control female mice was comparable to that of GABA-treated females (Figure 3D). At the end of the 16 weeks, the bodyweight of control females was not significantly different to that of GABA-treated females (Figure 3E). Extended analyses showed that during the treatment period, the control females had bodyweight gains similar to those of the GABA-treated females (Figure 3F).

**Figure 4:**
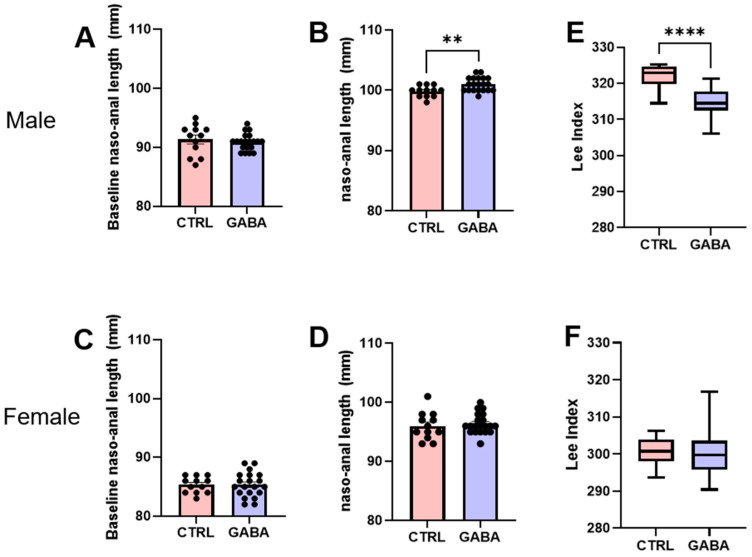
GABA supplementation enhances the growth of body length and decreases Lee index in male mice but not of females. (**A**) Baseline nasoanal length of control (91.33 ± 0.74 mm, n = 12) and GABA-treated males (90.90 ± 0.32 mm, n = 20). (**B**) Nasoanal length of GABA-treated males compared to controls after the 16-week treatment (control: 99.83 ± 0.27 mm, n = 12; GABA-treated: 101.0 ± 0.25 mm, n = 20, *p* < 0.01). (**C**) Pre-treatment nasoanal lengths of control females compared to the GABA-treated group (control: 85.33 ± 0.40 mm, n = 12; GABA-treated: 85.35 ± 0.47 mm, n = 20). (**D**) Post-treatment nasoanal length of control and GABA-treated females (control: 96 ± 0.67 mm, n = 12; GABA-treated: 96.4 ± 0.38 mm, n = 20). (**E**) Box and whisker plots of the Lee index of GABA-treated male mice compared to controls in arbitrary units (control: 321.7 ± 1.03, n = 12; GABA-treated: 314.7 ± 0.85, n = 20; *p* < 0.0001). (**F**) Lee index of control and GABA-treated females (control: 300.7 ± 1.07, n = 12; GABA-treated females: 300.4 ± 1.52, n = 20). Lee index is calculated in arbitrary units. Statistical significance indicated as ** *p* < 0.01, **** *p* < 0.0001.

As shown in Figure 4A, the baseline nasoanal length of control male mice was comparable to that of the GABA-treated males. After the 16-week treatment, the nasoanal length of GABA-treated males was significantly longer than that of control males (Figure 4B). Notably, over the treatment period, the total linear growth gain of GABA-treated males was about 15% greater than that of the control males.

The control and treated females had comparable baseline nasoanal lengths (Figure 4C). In contrast to male mice, at the end of the 16-week period, the nasoanal length of controls was not significantly different from that of GABA-treated females (Figure 4D). The lack of effect by GABA supplementation in female mice was clearer by examining the increase in length over the treatment period, which had no significant difference between control and treated females.

The Lee index of body fat, a function of bodyweight and body length, has been widely used to estimate body fat in normal and obese mice [29]. We therefore calculated the Lee index to estimate the effect of GABA supplementation on mouse body fat after the 16-week treatment. Remarkably, the Lee index of GABA-treated male mice was significantly lower than that of control males (Figure 4E). However, the Lee index of GABA-treated females was not significantly different from that of control females (Figure 4F). Together, these results suggest that long-term supplementation of GABA sex-dependently attenuates body-fat-mass gain during the adolescent growth period.

### 3.3. Effects of GABA on Metabolism and Locomotor Activity

**Figure 5:**
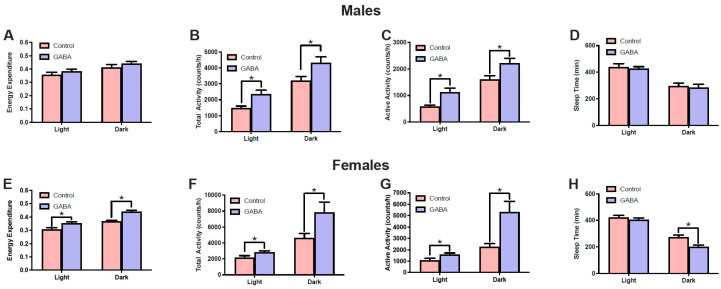
GABA supplementation exerts sex-specific effects on metabolism and locomotor activity. (**A**) Post-treatment energy expenditure between control and GABA-treated male mice during the light (control: 0.356 ± 0.018, n = 8; GABA-treated: 0.384 ± 0.015, n = 8) and dark (control: 0.413 ± 0.022, n = 8; GABA-treated: 0.440 ± 0.017, n = 8) phases. (**B**) Total locomotor activity in GABA-treated and control mice during the light (control: 1484 ± 117, n = 8; GABA-treated: 2357 ± 246, n = 8; *p* = 0.0064) and dark (control: 3212 ± 252, n = 8; GABA-treated: 4322 ± 375, n = 8; *p* = 0.0278) phases. (**C**) Active activity among control and GABA-treated males during the light (control: 593 ± 45, n = 8; GABA-treated: 1121 ± 151, n = 8; *p* = 0.0048) and dark (control: 1600 ± 148, n = 8; GABA-treated: 2214 ± 187, n = 8; *p* = 0.0221) phases. (**D**) Sleep duration between GABA-treated males and controls during the light (control: 439 ± 26, n = 8; GABA-treated: 427 ± 15, n = 8) and dark (control: 296 ± 23, n = 8; GABA-treated: 285 ± 24, n = 8) phases. (**E**) Energy expenditure among GABA-treated females during the light (control: 0.306 ± 0.013, n = 8; GABA-treated: 0.354 ± 0.011, n = 8; *p* = 0.0138) and dark (control: 0.369 ± 0.007, n = 8; GABA-treated: 0.440 ± 0.011, n = 8; *p* < 0.0001) phases. (**F**) Total activity among GABA-treated and control females during the light (control: 2158 ± 0.244, n = 8; GABA-treated: 2840 ± 189, n = 8; *p* = 0.044) and dark (control: 4627 ± 545, n = 8; GABA-treated: 7827 ± 189, n = 8; *p* = 0.041) phases. (**G**) Active activity between control and GABA-treated females during the light (control: 1073 ± 175, n = 8; GABA-treated: 1581 ± 134, n = 8; *p* = 0.0368) and dark (control: 2251 ± 292, n = 8; GABA-treated: 5330 ± 924, n = 8; *p* = 0.0067) phases. (**H**) Sleep time during the dark (control: 273 ± 16, n = 8; GABA-treated: 202 ± 12, n = 8; *p* = 0.0039) and light (control: 422 ± 16, n = 8; GABA-treated: 404 ± 15, n = 8) phases among GABA-treated females and controls. Statistical significance indicated as * *p* < 0.05.

The sex-dependent effects of GABA on food consumption and Lee index implied metabolic or behavioral changes in these mice. Therefore, we examined the metabolism and locomotor activities in both male and female mice using CLAMS. Results showed that GABA treatment induced a slight but not statistically significant increase in energy expenditure in male mice under both light and dark phases (Figure 5A). Female mice generally have a lower absolute energy expenditure rate compared to male mice [38,39]. Interestingly, GABA treatment significantly increased energy expenditure in GABA-treated female mice compared to control females (Figure 5E). This effect was more prominent during the dark phase but was also present in the light phase.

Thermogenic energy expenditure is closely related to locomotor activity. Analyses showed that both the total activity and active activity were significantly increased in GABA-treated male mice compared to control males during both light and dark phases (Figure 5B,C). GABA treatment also significantly increased the total activity and active activity in female mice compared to controls (Figure 5F,G). Active activity during the dark phase showed the most dramatic difference, with GABA-treated females having approximately 100% greater active activity than controls.

Typically, mice have reduced sleep duration during the dark phase compared to the light phase. GABA treatment did not significantly affect sleep times in male mice (Figure 5D) but significantly reduced the sleep times of GABA-treated female mice in the dark phase compared to control mice (Figure 5H).

### 3.4. Effects of GABA on Pancreatic β-Cell Mass and Blood Glucose

**Figure A1:**
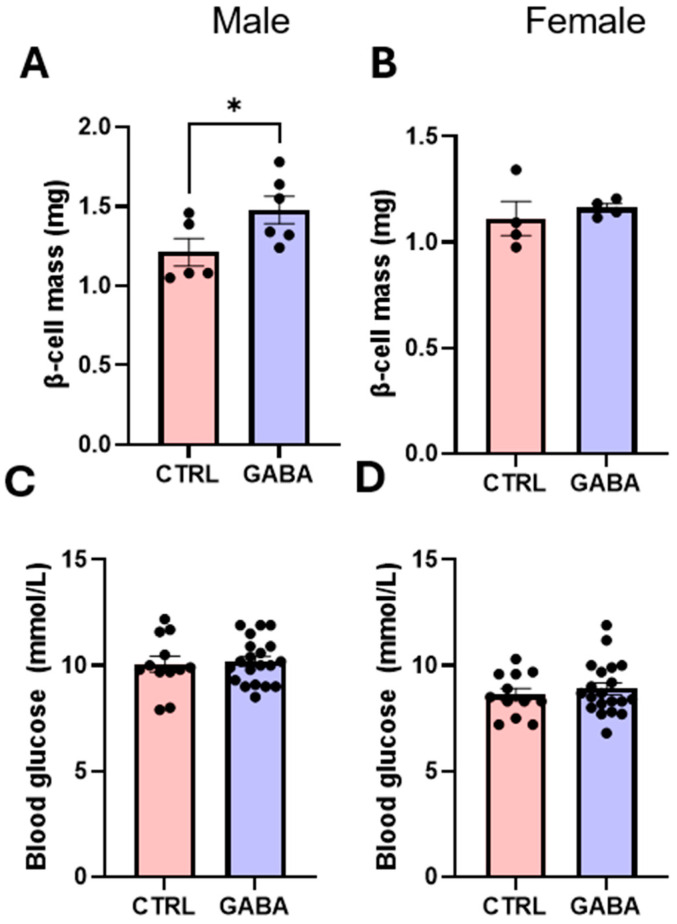
GABA treatment increases pancreatic β-cell mass in males but does not affect blood glucose concentrations in either sex. (**A**) Pancreatic β-cell mass among control and GABA-treated male mice (control: 1.21 ± 0.09 mg, n = 5; GABA-treated: 1.48 ± 0.09 mg, n = 6, *p* < 0.05). (**B**) β-cell mass between treated and untreated female mice (control: 1.11 ± 0.08 mg, n = 4; GABA-treated: 1.16 ± 0.02 mg, n = 4, *p*= 0.28). (**C**) Post-treatment blood glucose concentrations in male control and GABA-treated mice (control: 10.07 ± 0.38 mmol/L, n = 12; GABA-treated: 10.20 ± 0.24 mmol/L, n = 20, *p* = 0.75). (**D**) Blood glucose concentrations among GABA-treated and untreated females (control: 8.61 ± 0.30 mmol/L, n = 12; GABA-treated: 8.90 ± 0.28 mmol/L, n = 20, *p* = 0.51). Statistical significance indicated as * *p* < 0.05.

GABA metabolism through the GABA shunt has been shown to play a role in the oxidative phosphorylation of glucose [40]. In addition, long-term ad libitum administration of GABA in healthy adult mice increased pancreatic β-cell mass and caused a modest enhancement in insulin secretion [41]. Indeed, immunohistochemical analyses in this study showed that oral administration of GABA modestly but significantly increased the pancreatic β-cell mass of male mice (Appendix A, Figure A1A) but not females (Appendix A, Figure A1B). Consistent with a study in healthy human volunteers [42], however, supplementation of GABA did not change the blood glucose concentrations of either male or female mice (Appendix A, Figure A1C,D).

### 3.5. Effects of GABA on Lipolytic Enzyme Activation in Adipose Tissue

**Figure 6:**
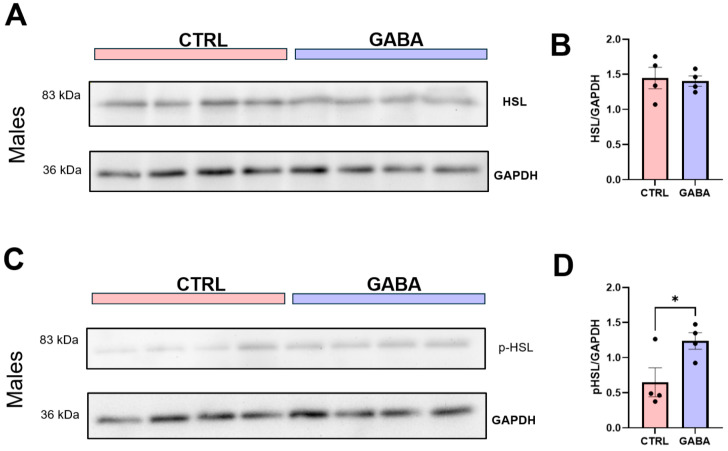
GABA treatment increases phosphorylation of HSL in adipose tissues of male mice. (**A**) Representative immunoblots of HSL and GAPDH (loading control) in adipose tissue from control and GABA-treated male mice. (**B**) Bar graph displaying HSL expression normalized to GAPDH between control and GABA-treated males (control: 1.45 ± 0.15, n = 4; GABA-treated: 1.403 ± 0.07, n = 4). (**C**) Representative blot of *p*-HSL and GAPDH expression in adipose tissues. (**D**) Normalized *p*-HSL expression in adipose tissue of male GABA-treated mice compared to controls (control: 0.65 ± 0.21, n = 4; GABA-treated: 1.24 ± 0.12, n = 4; *p* < 0.05). Data measured in arbitrary units by densitometry. Statistical significance indicated as * *p* < 0.05.

Given the observed effects of GABA treatment on the Lee index of male mice, which indicated lower body fat content in GABA-treated males, this study explored whether GABA treatment affected the key lipolytic enzyme hormone-sensitive lipase (HSL) in adipose tissues of these mice. Immunoblot analyses of gonadal adipose tissue revealed no significant differences in the relative expression of hormone-sensitive lipase (HSL) between controls and GABA-treated male mice (Figure 6A,B). However, GABA-treated males expressed a significantly greater amount of phosphorylated HSL (*p*-HSL) (Figure 6C,D), indicating greater activation of this lipolytic enzyme in the GABA-treated males.

### 3.6. Effects of GABA on Serum GH, Pituitary GH, and GH-Positive Cells

**Figure 7:**
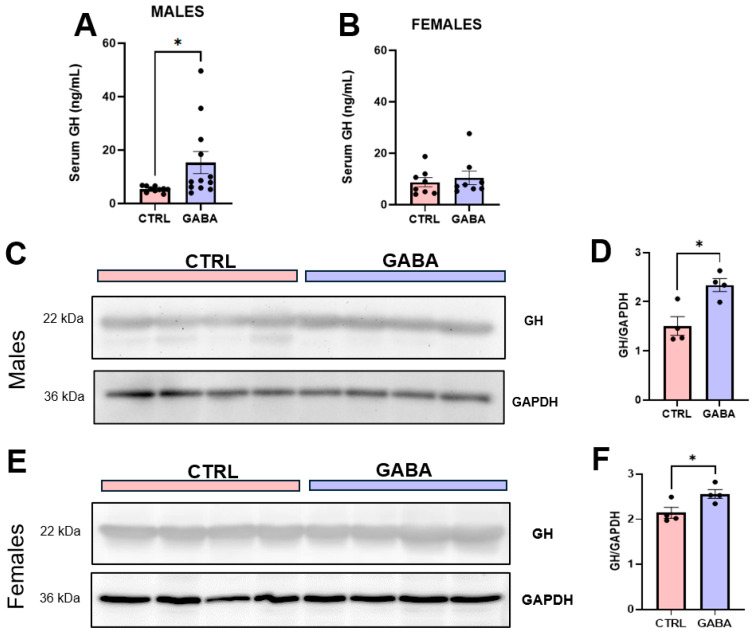
GABA supplementation significantly increases the levels of GH in the sera and pituitary of male mice. (**A**) Serum GH concentrations in GABA-treated male mice compared to controls (control: 5.37 ± 0.34 ng/mL, n = 10; GABA-treated: 15.29 ± 4.14 ng/mL, n = 12; *p* < 0.05). (**B**) Serum GH concentration among GABA-treated and control females (control: 8.73 ± 1.77 ng/mL, n = 8; GABA-treated: 10.42 ± 2.66 ng/mL, n = 8; *p* = 0.61). (**C**) Representative immunoblots of GH protein and the loading control GAPDH in the pituitaries of control and GABA-treated males. (**D**) Bar graph displays the ratio of GH/GAPDH between GABA-treated males and controls (control: 1.50 ± 0.19, n = 4; GABA-treated: 2.34 ± 0.13, n = 4; *p* < 0.05). (**E**) Representative immunoblots of GH protein and GAPDH in the pituitaries of control and GABA-treated females. (**F**) Normalized GH protein in the pituitary of GABA-treated females compared to controls (control: 2.14 ± 0.12, n = 4; GABA-treated: 2.56 ± 0.10, n = 4; *p* < 0.05). Western blot data measured in arbitrary units by densitometry. Statistical significance indicated as * *p* < 0.05.

Given that growth hormone (GH) increases food intake [43], stimulates lipolysis in adipocytes [44], increases energy expenditure [45], and reduces body fat mass [46], we examined whether GABA supplementation affected GH concentration in serum. Results from ELISA revealed that GABA treatment significantly increased the level of GH in the sera of male mice (Figure 7A) but not the females (Figure 7B). Furthermore, the baseline level of serum GH was notably higher in the female mice than that in male mice (Figure 7A,B). Remarkably, immunoblot assays showed that GH protein expression in the pituitary was significantly increased in GABA-treated male mice (Figure 7C,D) and in GABA-treated female mice (Figure 7E,F) compared to their respective controls.

**Figure A2:**
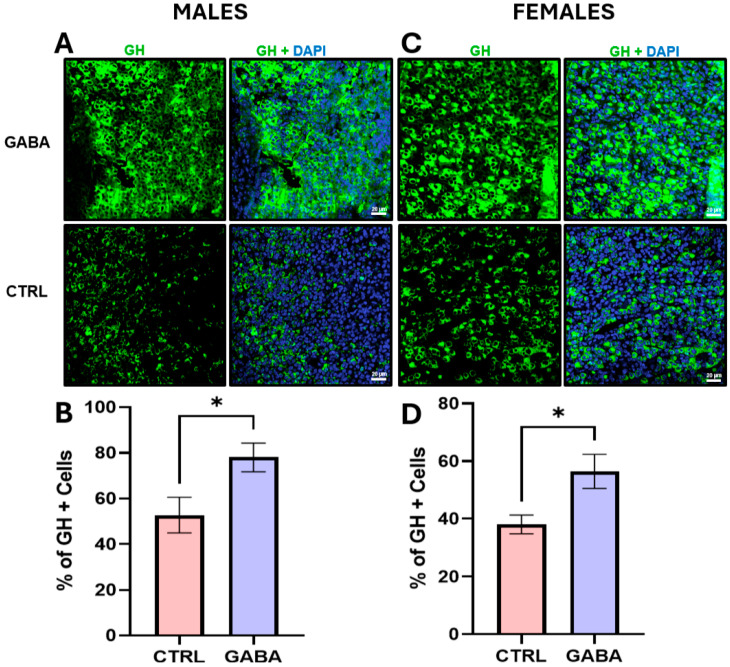
GABA supplementation increases the GH+ cell proliferation in male and female mice. (**A**) Representative immunofluorescence images of GH-positive cells (green) in the coronal pituitary sections of GABA-treated male mice (upper row) and control male mice (lower row). DAPI (blue) staining shows the total cell populations. (**B**) Bar graph representing the percentage of GH-positive cells in the pituitaries of GABA-treated and control males (control: 52.67 ± 7.78%, n = 4; GABA-treated males: 77.82 ± 6.36%, n = 4; *p* < 0.05). (**C**) Representative immunofluorescence images of GH-positive cells (green) and DAPI staining (blue) in the pituitary of GABA-treated female mice (upper row) and control females (lower row). (**D**) Percentage of GH-positive cells among control and GABA-treated females (control: 38.07 ± 3.26%, n = 4; GABA-treated females: 56.45 ± 5.92%, n = 4; *p* < 0.05). Statistical significance indicated as * *p* < 0.05.

**Figure A3:**
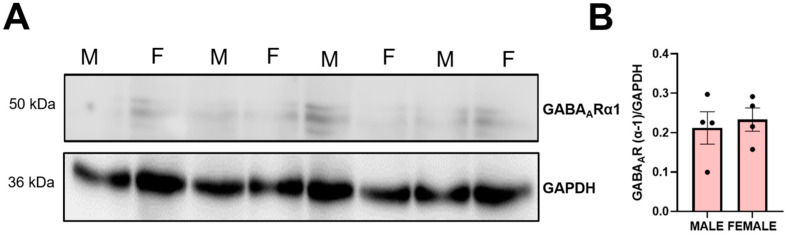
(**A**) No sex differences in GABA_A_R expression. Representative Western blot of GABA_A_R (α1 subunit) and GAPDH loading control in untreated male and female mice. (**B**) Bar graph showing normalized GABA_A_R expression between male and female untreated mice (males: 0.21 ± 0.04, n = 4; females: 0.23 ± 0.30, n = 4; *p* = 0.69). Measured in arbitrary units by densitometry.

In addition, immunohistochemical analyses of coronal pituitary sections revealed that compared to control males, GABA-treated males displayed an approximately 30% increase in GH-immune-positive (GH+) cells in the total pituitary cell populations (Appendix A, Figure A2A,B). Similarly, GABA-treated females also displayed a higher proportion of GH+ cells (Appendix A, Figure A2C,D). Previous studies showed that GABA_A_Rs are expressed in the anterior pituitary [47]. Immunoblot analysis revealed no differences in GABA_A_R expression between male and female mice (Appendix A, Figure A3A,B).

### 3.7. Effects of GABA on GH Downstream Effectors in the Liver

**Figure 8:**
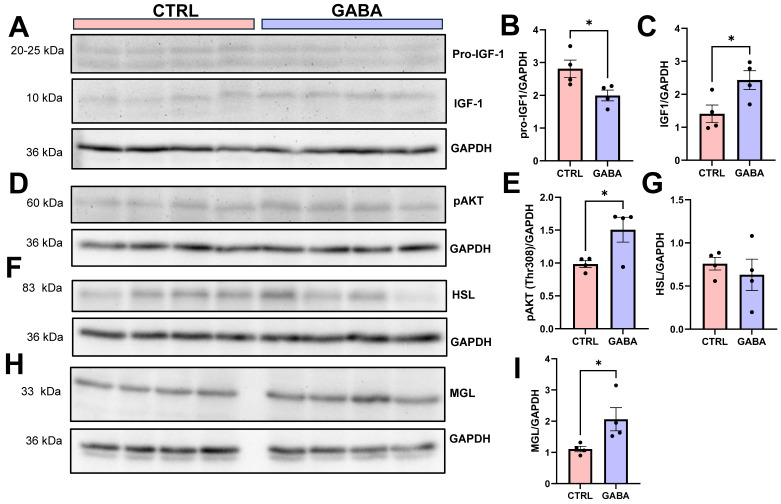
Effect of GABA supplementation on GH downstream effectors in the liver of male mice. (**A**) Representative immunoblots of pro-IGF1, mature IGF1, and GAPDH loading control in whole liver lysates from control and GABA-treated male mice. (**B**) Bar graph showing the normalized expression of pro-IGF1 in GABA-treated and control males (control: 2.81 ± 0.26, n = 4; GABA-treated: 2.00 ± 0.16, n = 4; *p* < 0.05). (**C**) Normalized expression of mature IGF1 in GABA-treated males compared to controls (control: 1.41 ± 0.27, n = 4; GABA-treated: 2.43 ± 0.29, n = 4; *p* < 0.05). (**D**) Representative blot of *p*-AKT and GAPDH expression in the livers of control and GABA-treated male mice. (**E**) Normalized *p*-AKT expression among control and GABA-treated males (control: 0.98 ± 0.05, n = 4; GABA-treated: 1.51 ± 0.19, n = 4; *p* < 0.05). (**F**) Representative blot of liver HSL and GAPDH expression in control and treated male mice. (**G**) HSL expression after GABA treatment between control and GABA-treated males (control: 0.76 ± 0.07, n = 4; GABA-treated: 0.63 ± 0.18, n = 4). (**H**) Representative blot of MGL and GAPDH in the livers of control and GABA-treated males. (**I**) MGL expression among GABA-treated male mice compared to controls (control: 1.11 ± 0.09, n = 4; GABA-treated: 2.06 ± 0.37, n = 4; *p* < 0.05). Data measured in arbitrary units by densitometry. Statistical significance indicated as * *p* < 0.05.

The results of this study revealed greater expression and secretion of GH with concurrent increases in linear growth and food intake in male GABA-treated mice. Therefore, it was important to further investigate if the effects of GABA treatment occurred via GH and its major downstream effector insulin-like growth factor 1 (IGF1) in the liver, a major organ of glucose and lipid metabolism. Western blot analyses of whole liver tissues revealed a significantly lower expression of IGF1 precursor (pro-IGF1) with a corresponding greater expression of mature IGF1 in GABA-treated males compared to controls (Figure 8A–C). Moreover, the liver of GABA-treated males expressed greater phosphorylated AKT (*p*-AKT) (Figure 8D,E), a key mediator of the IGF1 signaling pathway. Interestingly, GABA treatment did not change the level of HSL (Figure 8F) but significantly increased the level of monoacylglycerol lipase (MGL) as shown in Figure 8H,I. MGL is a key enzyme in hydrolyzing intracellular triglyceride stores to fatty acids and glycerol [48]. Together our results strongly suggest that GABA treatment increased circulating GH, which in turn enhances growth and lipolysis by activating its downstream signaling and upregulating lipolytic enzymes.

### 3.8. Effects on Hypothalamic GHRH and Gastric Ghrelin

**Figure A4:**
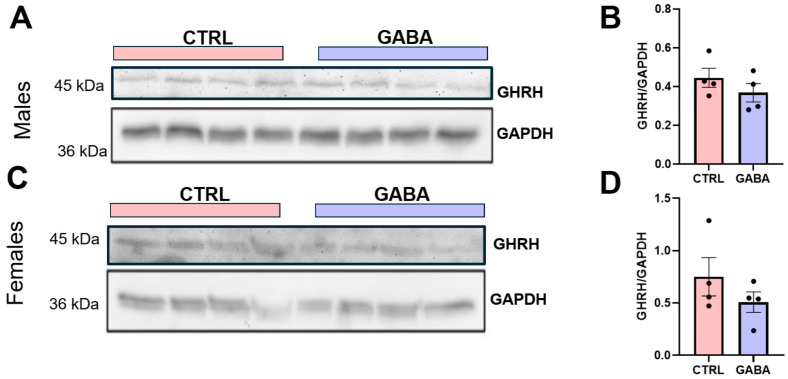
GABA supplementation does not significantly change the expression level of GHRH in the hypothalamus of male and female mice. (**A**) Representative immunoblots of pre-pro GHRH and GAPDH (loading control) in the hypothalamus of male control and GABA-treated mice. (**B**) Bar graph analysis of hypothalamic pre-pro GHRH normalized to GAPDH in male GABA-treated and untreated mice (control: 0.45 ± 0.05, n = 4; GABA-treated: 0.37 ± 0.05, n = 4; *p* = 0.31). (**C**) Representative blot of pre-pro GHRH and GAPDH in female treated and untreated mice. (**D**) Bar graph of normalized pre-pro GHRH expression in control and GABA-treated females (control: 0.75 ± 0.18, n = 4; GABA-treated: 0.51 ± 0.10, n = 4; *p* = 0.29).

How did GABA treatment increase pituitary GH expression and secretion? GH secretion is essentially controlled by hypothalamic growth hormone-releasing hormone (GHRH) [49] and by ghrelin, which is primarily secreted from the stomach epithelium [50], via the growth hormone-releasing hormone receptors (GHRH-Rs) and growth hormone secretagogue receptors (GHS-Rs), respectively. Immunoblot analyses showed that GABA supplementation did not significantly change the expression level of GHRH in the hypothalamus of both male mice (Appendix A, Figure A4A,B) and female mice (Appendix A, Figure A4C,D). These results might reflect the nature of GABA molecules, which under normal conditions are unable to cross the blood–brain barrier (BBB) in functionally significant quantities [51].

**Figure 9:**
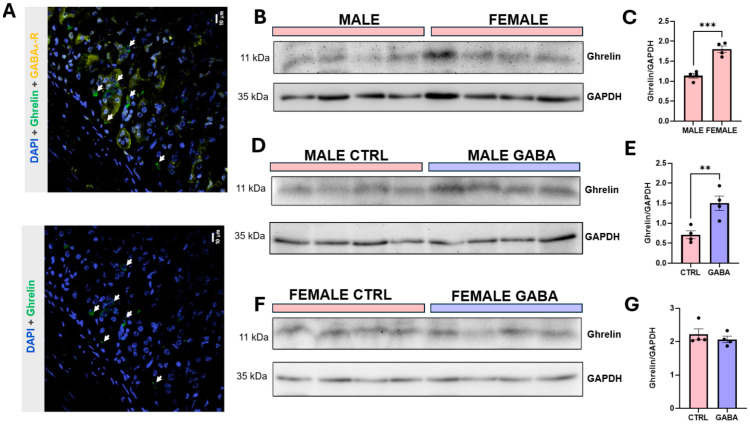
GABA supplementation increases the level of ghrelin in the stomach of male mice but not females. (**A**) Upper image shows immunofluorescence of the α1-subunit of the GABAARs (yellow), and ghrelin (green) and DAPI staining (blue). Lower image shows immunofluorescences of ghrelin and DAPI staining. Arrows represent ghrelin colocalized with GABAARs. (**B**) Representative immunoblots of ghrelin protein and GAPDH (loading control) in the stomach of non-treated males and females. (**C**) Bar graph displays ghrelin normalized to GAPDH in control females and males (males: 1.14 ± 0.06, n = 4; females: 1.80 ± 0.08, n = 4, *p* < 0.001). (**D**) Representative immunoblots of ghrelin protein and GAPDH in control and GABA-treated males. (**E**) Normalized ghrelin expression in control and GABA-treated males (control: 0.71 ± 0.10, n = 4; GABA-treated: 1.50 ± 0.18, n = 4, *p* < 0.01). (**F**) Representative immunoblots of ghrelin protein and GAPDH in control and GABA-treated females. (**G**) Normalized ghrelin expression among GABA-treated and untreated females (control: 2.22 ± 0.16, n = 4; GABA-treated: 2.07 ± 0.10, n = 4, *p* = 0.46). Data are measured in arbitrary units by densitometry. Statistical significance indicated as ** *p* < 0.01, *** *p* < 0.001.

**Figure A5:**
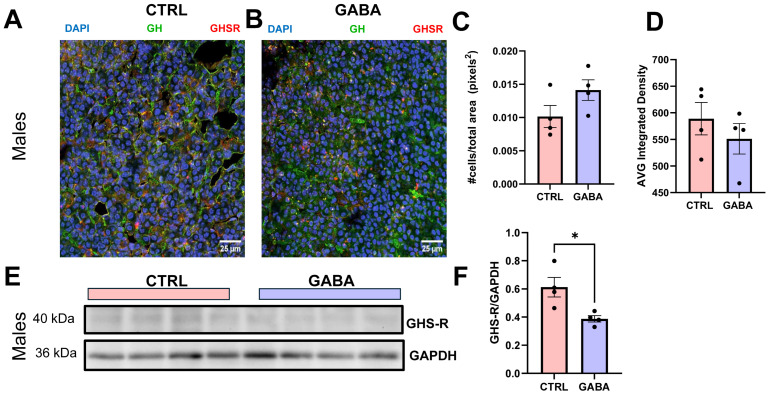
Effects of GABA treatment on GHS-R expression in male mice. The immunohistochemical analyses show the expression of GHS-Rs (red) in GH+ cells (green) in coronal pituitary sections of control (**A**) and GABA-treated (**B**) male mice, with DAPI staining (blue) to visualize the cell density. (**C**) Bar graph showing the cell density of GHS-R-positive cells in the pituitaries of male treated and untreated mice (control: 0.010 ± 0.002, n = 4; GABA-treated: 0.014 ± 0.002, n = 4; *p* = 0.13). (**D**) Bar graph showing GHS-R immunofluorescence intensity in pituitary sections of control and GABA-treated males (control: 588.9 ± 30.55, n = 4; GABA-treated: 551.2 ± 28.69, n = 4; *p* = 0.40). (**E**) Representative Western blot of GHS-R and GAPDH (loading control) in whole pituitary lysate of male treated and untreated mice. (**F**) Normalized GHS-R protein expression in the pituitaries of control and GABA-treated male mice (control: 0.61 ± 0.07, n = 4; GABA-treated: 0.39 ± 0.02, n = 4; *p* < 0.05). Statistical significance indicated as * *p* < 0.05.

On the other hand, our novel findings from immunohistochemical assays showed that the α1-subunit of the GABA_A_R was expressed in ghrelin-immune-positive (Ghrelin+) cells of mouse stomach epithelia (Figure 9A). Immunoblot assays showed that the baseline expression level of ghrelin in the stomach of male mice was significantly lower than that of female mice (Figure 9B,C). Notably, GABA treatment significantly increased the ghrelin expression in the stomachs of male mice (Figure 9D,E) but not female mice (Figure 9F,G). Our immunohistochemical analyses, displayed in Appendix A, Figure A5A,B, showed that GABA supplementation did not significantly affect the number of GHS-R-expressing cells (Appendix A, Figure A5C) or the GHS-R antibody fluorescence intensity (Appendix A, Figure A5D) in the pituitary of male mice. Yet, immunoblot assays showed that GABA supplementation decreased the expression of GHSR in the pituitary of male mice (Appendix A, Figure A5E,F). Together, these results suggested that GABA supplementation increases GH secretion from the pituitary by increasing ghrelin production and/or secretion.

## 4. Discussion

GABA is marketed in the U.S. and Canada as a dietary supplement. The present study demonstrates that long-term (16 weeks) ad libitum supplementation of GABA to young growing mice has profound sex-dependent impacts on adolescent development. First, GABA supplementation significantly increases food consumption and body length growth but suppresses bodyweight gain, consequently lowering the Lee index in male but not female mice. Second, supplementing GABA increases locomotor activity in both sexes but only facilitates lipolysis in male mice. Third, and importantly, GABA administration increases circulating GH and gastric ghrelin in males but not females. These novel findings suggest that long-term supplementation of GABA causes significant impacts on endocrine functions, thereby modulating lipid metabolism, body growth, and motor activity during the developmental period from early adolescence to young adulthood.

In the present study, GABA was administered to young mice through drinking water, similar to previous studies in various animal models of disease [10,20], because in this way, GABA could be readily and stably supplemented to the animals without causing stress. Importantly, the average weekly dose of GABA to a test mouse could be estimated from water consumption. To mimic nutrient supplementation, the administered dose of GABA in this study was lower than that in many previous studies, although it is reported by the United States Pharmacopeia (USP) that long-term oral administration of GABA even at much larger doses does not cause toxic effects [52].

Oral GABA from natural foods or by supplementation is absorbed via the activity of GABA transporters in the apical membrane of gastrointestinal epithelial cells [53] and then routed through the portal vein into the liver, where most GABA molecules are transported into hepatocytes [54], and the rest enters the systemic circulation. It has been demonstrated that ad libitum supplementation of GABA to mice significantly increases the concentration of GABA in the circulation [42]. Given that under physiological conditions, GABA influx across the BBB is minimal compared to the much greater efflux [51], we postulate that in this study, the observed effects of GABA supplementation primarily result from its actions on various cells in peripheral tissues. Considering that GABA supplementation increases the production and secretion of a range of hormones including GH and ghrelin, which can pass the BBB and act on neurons in the hypothalamus, thus synergically increasing food intake [55,56], we propose that the GABA-induced changes in food consumption and sleep are indirect actions in the central nervous system via peripherally secreted hormones.

Studies on several different species such as lambs [57], cows [58], and even chickens [59] have found that GABA supplementation can stimulate food intake. However, few previous studies investigated the effect of oral administration of GABA on food consumption in young mice undergoing growth. One study reported that including a high dose (5%) of GABA in food decreased the food intake by lean IRC mice [60]. In contrast, the present study found that only a week after adding low-dose GABA to the drinking water, there was a 6% increase in food intake among male mice but not females, and such an increase in food consumption by male mice persisted during the 16-week treatment period. Considering the consistently higher food intake during the treatment period, the 6% increase in food consumption is a significant amount. The difference in GABA effects on food intake between the two studies may result from the different doses of GABA and the method of administration (in water vs. food), as well as differences in mouse strains.

A novel and interesting finding from this study was that supplementing GABA increased body length growth but decreased the bodyweight gain, resulting in lower Lee index values in male mice during development under a “standard” low-fat diet. Paradoxically, these GABA-treated male mice showed higher food consumption but lower weight gain and body fat index. These seemingly divergent results suggest that the behavioral or metabolic effect of GABA is responsible for the reduction in body fat. Early studies reported that GABA reduces infiltration of M1-like macrophages into the subcutaneous white adipose tissue [22] and promotes beige adipocyte reconstruction [19]. More recently, a study reported that oral administration of a high dose of GABA to mice fed with a high-fat diet effectively reduced the amount of lipids and lipid proteins in circulation, thus suppressing adipogenesis [20]. Interestingly, our analyses demonstrated that supplementing GABA reduced the fat mass index and increased lipolytic enzymes in adipose tissues of adolescent male mice fed with a “standard” low-fat diet. Notably, GABA supplementation to these healthy mice had no effect on their blood glucose, consistent with a previous clinical trial in which the administration of GABA to healthy subjects did not change the level of blood glucose [61]. Moreover, metabolic analyses showed that female mice were generally more active than their male counterparts. Yet, GABA administration greatly augmented locomotor activity in both sexes, although it only significantly reduced the sleep times and increased the thermogenic energy expenditure in female mice. Together, these results imply that GABA supplementation induces sex-dependent alterations in growth, lipid metabolism, locomotion, and energy expenditure.

How does GABA regulate growth and lipid metabolism? Early studies reported that ghrelin and GH increase food intake and facilitate body growth [55,56], and sex-dependently regulate lipid metabolism [62,63]. In addition, type-A receptors (GABA_A_Rs) are expressed in several types of endocrine cells including GH-producing cells in the anterior lobe of the pituitary [64], and activation of GABA_A_Rs in pituitary cells results in membrane depolarization and calcium entry via voltage-gated calcium channels [47]. We therefore examined whether long-term supplementation of low-dose GABA constantly increased the expression and secretion of GH. Previous studies reported that ingestion of large doses of GABA caused a rapid but short-term stimulation of blood GH concentration in rats [65] and humans [66]. However, the effect of long-term GABA supplementation on pituitary GH expression and secretion had not been well examined. Indeed, the present study found that long-term GABA supplementation significantly increased GH in the serum of male mice but not females. Yet, our further analyses showed that GABA supplementation significantly increased the expression level of pituitary GH in both male and female mice.

Considering that the secretion of GH is tightly controlled by GHRH [49] and ghrelin [67], we also examined the effects of GABA treatment on the expression levels of GHRH in the hypothalamus and ghrelin in the stomach of tested mice accordingly. Our results showed that GABA supplementation had no effect on the expression of GHRH, which may signify that supplemented GABA does not enter the brain under physiological conditions, or at least not in sufficient amounts to affect hypothalamic GHRH neurons. On the other hand, analyses of stomach tissues showed that GABA supplementation significantly increased ghrelin expression in male mice. Ghrelin is well known to stimulate GH secretion [68,69]. Specifically, it is a stimulator of pituitary-specific transcription factor (Pit-1) [70]. Pit-1 activates transcription on the GH gene and is therefore essential for GH synthesis in somatotrophs [71]. Given the observed increases in ghrelin and GH expression among male GABA-treated mice in this study, it is plausible that GABA supplementation increased pituitary and blood GH indirectly via an upregulation of systemic ghrelin, which promotes GH synthesis and secretion. These findings indicate that the GABA-induced increase in GH secretion among males is at least partly associated with increases in the production and secretion of ghrelin. Aside from stimulating feeding and promoting GH secretion, ghrelin is strongly associated with increases in locomotor activity via its actions in the CNS [72,73,74] and may act on central neurons synergistically with GH to increase caloric expenditure.

It has been well established that GH stimulates lipolysis in adipocytes [44] and is associated with reductions in body fat mass [46]. Indeed, our results showed that GABA treatment not only increased circulating GH levels but also increased pHSL, an activated form of this lipolytic enzyme, in adipose tissue. Our immunoblotting analysis of liver tissues further demonstrated that in male mice, GABA treatment increased the GH downstream factors IGF1 and pAKT, which critically regulate hepatic function and lipid metabolism [75,76]. Increased lipolysis in adipose tissues raises the levels of free fatty acids and monoglycerides in circulation, which may place a higher demand for the liver to break these down, resulting in higher expression of lipolytic enzymes such as MGL [77]. Together, results from the present study indicate that supplementation of GABA to juvenile mice lowered the Lee index by upregulating the expression and secretion of ghrelin and GH which promotes lipolysis in cells including adipocytes and hepatocytes. More specifically, they are indicative that the observed attenuation of weight gain in male mice is likely driven by GH-induced increases in lipolysis of adipose tissue as well as contributions from the higher locomotor activity observed in these mice. This increase in activity level, as well as greater food consumption, is probably due to GABA-induced increases in circulating ghrelin among male mice.

GABA supplementation caused more significant effects on growth and food intake in males whereas it had larger effects on locomotor activity and energy expenditure in females. These sex-dependent effects of GABA supplementation may be related to its sex-dependent regulation of serum GH, pituitary GH, and gastric ghrelin, which were notably higher in control female mice than control males. In this regard, previous studies demonstrated that GH sex-dependently regulates metabolism. Specifically, males are more responsive to the lipolytic effects of GH as measured by blood markers of lipid metabolism [78]. Males also exhibit more pronounced changes in body composition and greater serum IGF-1 response to GH treatment [79,80]. Moreover, testosterone has been shown to increase levels of IGF-1 [81], which may have a synergistic effect with the GABA-induced increase in GH secretion, while estrogen has the opposite effect [82]. Similarly, the regulatory effects of ghrelin on feeding and locomotor activity are different between males and females. Basal levels of ghrelin are higher in females than in males, which may partially explain why males are more responsive to the ghrelin-stimulating effects of supplemented GABA [83]. Male and ovariectomized female rats are more sensitive to the appetite-stimulating effects of peripherally administered ghrelin [84]. Furthermore, ghrelin deficiency in a binge-eating mouse model caused a stronger reduction in activity levels in females, suggesting that ghrelin plays a more critical role in regulation of locomotor activity in females [74]. Nevertheless, the sex-dependent effects of GABA supplementation await further studies.

## 5. Conclusions

GABA receptors are widely expressed in many types of non-neuronal cells including GH-producing cells [64] and ghrelin-producing cells (Figure 9A). Studies on the cellular and molecular mechanisms by which GABA directly or indirectly upregulates the production and secretion of ghrelin and GH are currently ongoing in our laboratory. The reported effects of GABA supplementation on sleep [85], anxiety [86], and cognition [87] lead to considerations of whether circulating GABA may enter the brain through the BBB, thereby exerting direct central effects. Early research on this matter found no BBB permeability to GABA [88], while more recent results demonstrated the activity of GABA transporters at the BBB [89]. However, as stated earlier, GABA efflux across the BBB is 16 times greater than the influx [51], making it unlikely to accumulate in the brain in functionally significant quantities. Moreover, intravenous drip of GABA in rats was found to have no significant effect on brain GABA concentrations [90]. Although issues as to whether the peripherally administered GABA can cross the BBB under normal conditions remain controversial, the regulatory effects of GABA on the function of certain peripheral cells are often fully attributed to direct activation of GABA receptors in these cells. For example, a paper published in *Cell* journal in 2017 reported that long-term large doses of GABA to mice dramatically increased pancreatic beta cell mass by facilitating phenotypic transformation of alpha cells to beta cells [91] as these pancreatic endocrine cells express GABA receptors [92]. It is interesting that GABA and GH reportedly exert similar effects on diverse aspects of physiology or pathophysiology, including sleep [93], anxiety [94], cognitive function [95], growth [96], muscle mass gain [97], and lipid metabolism [20,46]. It is plausible that supplemented GABA exerts its central and peripheral effects not only through direct actions in target cells but also through various hormones, such as GH and ghrelin, which can readily cross the BBB and affect CNS function.

In summary, novel findings from this study demonstrate that the supplementation of GABA significantly upregulated both the levels of ghrelin and GH, thereby modulating food consumption, growth, locomotor behaviors, and lipid metabolism during the period of adolescence. Hormone-controlled growth and metabolism in early life critically affect the process of aging and the pathogenesis of diseases. Results from this study highlight the critical role of GABA in the regulation of development and the maintenance of physiological homeostasis during the adolescent period of growth. These findings indicate that while GABA supplementation may confer health benefits under certain conditions, caution is warranted, as prolonged GABA intake could influence endocrine function and alter metabolic processes. Further investigation is needed to elucidate the long-term effects of GABA supplementation on metabolic and hormonal regulation, particularly during early life.

## Data Availability

The raw data supporting the conclusions of this article will be made available by the authors upon request.
